# Exercise Training Attenuates Right Ventricular Remodeling in Rats with Pulmonary Arterial Stenosis

**DOI:** 10.3389/fphys.2016.00541

**Published:** 2016-12-05

**Authors:** Brunno Lemes de Melo, Stella S. Vieira, Ednei L. Antônio, Luís F. N. dos Santos, Leslie A. Portes, Regiane S. Feliciano, Helenita A. de Oliveira, José A. Silva, Paulo de Tarso C. de Carvalho, Paulo J. F. Tucci, Andrey J. Serra

**Affiliations:** ^1^Cardiac Physiology Laboratory, Federal University of São PauloSão Paulo, Brazil; ^2^Biophotonic Laboratory, Nove de Julho UniversitySão Paulo, Brazil

**Keywords:** artery pulmonary stenosis, cardiac hypertrophy, cardiac remodeling, exercise training, right ventricular hypertrophy

## Abstract

**Introduction:** Pulmonary arterial stenosis (PAS) is a congenital defect that causes outflow tract obstruction of the right ventricle (RV). Currently, negative issues are reported in the PAS management: not all patients may be eligible to surgeries; there is often the need for another surgery during passage to adulthood; patients with mild stenosis may have later cardiac adverse repercussions. Thus, the search for approaches to counteract the long-term PAS effects showed to be a current target. At the study herein, we evaluated the cardioprotective role of exercise training in rats submitted to PAS for 9 weeks.

**Methods and Results:** Exercise resulted in improved physical fitness and systolic RV function. Exercise also blunted concentric cavity changes, diastolic dysfunction, and fibrosis induced by PAS. Exercise additional benefits were also reported in a pro-survival signal, in which there were increased Akt_1_ activity and normalized myocardial apoptosis. These findings were accompanied by microRNA-1 downregulation and microRNA-21 upregulation. Moreover, exercise was associated with a higher myocardial abundance of the sarcomeric protein α-MHC and proteins that modulate calcium handling—ryanodine receptor and Serca 2, supporting the potential role of exercise in improving myocardial performance.

**Conclusion:** Our results represent the first demonstration that exercise can attenuate the RV remodeling in an experimental PAS. The cardioprotective effects were associated with positive modulation of RV function, survival signaling pathway, apoptosis, and proteins involved in the regulation of myocardial contractility.

## Introduction

Pulmonary arterial stenosis (PAS) is a common congenital heart disease that affects the RV outflow tract (Bonow et al., 2008; Tarasoutchi et al., 2011; Ananthakrishna et al., 2014), in which has been highly prevalent in women (Egbe et al., 2014). There is a consensus that patients with severe PAS are eligible for surgery intervention (e.g., balloon valvuloplasty), in which it shows to be implemented at an early age to prevent adverse RV remodeling. On the other hand, a mild RV obstruction is often only clinically supervised (Kan et al., 1982; Rey et al., 1988; Stanger et al., 1990). In this regard, a possible hypertrophy and functional RV abnormality is monitored in the long-term as a means to prevent advance to heart failure (Pokreisz et al., 2007; Kittipovanonth et al., 2008; Baumgartner et al., 2010). Although the current PAS clinical management has managed to correct the congenital defect and improve physiological RV variables, some weaknesses were reported. The surgical stenosis correction in early life is an important limitation in developing countries, in which late intervention may underlie the impaired RV remodeling, loss of functional capacity and reduced quality of life (Tchoumi et al., 2011; Romeih et al., 2013). It is common that rectifications of malformations affecting the RV outflow tract can result in valve regurgitation, restenosis, and later on it will need valve implantation (Ananthakrishna et al., 2014; Sizarov and Boudjemline, 2016). Moreover, data are showing that a maintenance of cardiac function is observed after invasive intervention without following evidence functional gain beyond the acute stage (Lurz et al., 2011). Further, an impaired RV systolic function can appear even after surgery repair in conditions of complex congenital defects (Khraiche and Ben Moussa, 2016). Ultimately, patients with mild PAS can have later RV remodeling, i.e., stenosis is exacerbated by body growth because of the limited size of the conduit. In the knowledge of the natural history of PAS, data are showing that mild cases can become severe at a later date (Anand and Mehta, 1997; Baumgartner et al., 2010).

Based on the limitations mentioned above, new approaches have been evaluated to counteract the long-term PAS effects. In an animal PAS model, Borgdorff and coworkers have shown that pharmacological treatment with Sildenafil was effective in attenuating RV dysfunction (Borgdorff et al., 2012). Considering that a lower exercise capacity is commonly seen in patients with RV hypertrophy (Meadows et al., 2007) or who had surgical repair during childhood (Roos-Hesselink et al., 2006), exercise training can be an attractive approach. Moreover, exercise has shown beneficial cardiac effects on models of left ventricular pressure-overload. In a recent study by Souza and coworkers, exercise training attenuated cardiac remodeling and preserved systolic and diastolic function in rats submitted to ascending aortic stenosis (Souza et al., 2015). In contrast, we found no data about the effects of exercise on RV pressure-overload hypertrophy because of mechanical obstruction to outflow. The only evidence comes from experimental studies carried out with RV pressure overload on pulmonary hypertension induced by monocrotaline. In this regard, exercise training was able to improve survival and cardiac function (Handoko et al., 2009).

In this study, we evaluated whether an aerobic exercise training would improve functional capacity and attenuate the RV remodeling in an experimental PAS model. We specifically hypothesized (i) that exercise would increase physical fitness as a safety approach, in other words, without adverse effects, and (ii) the findings obtained could be accomplished by attenuation of pathological RV remodeling.

## Materials and methods

### Animals and experimental design

**Figure 1:**
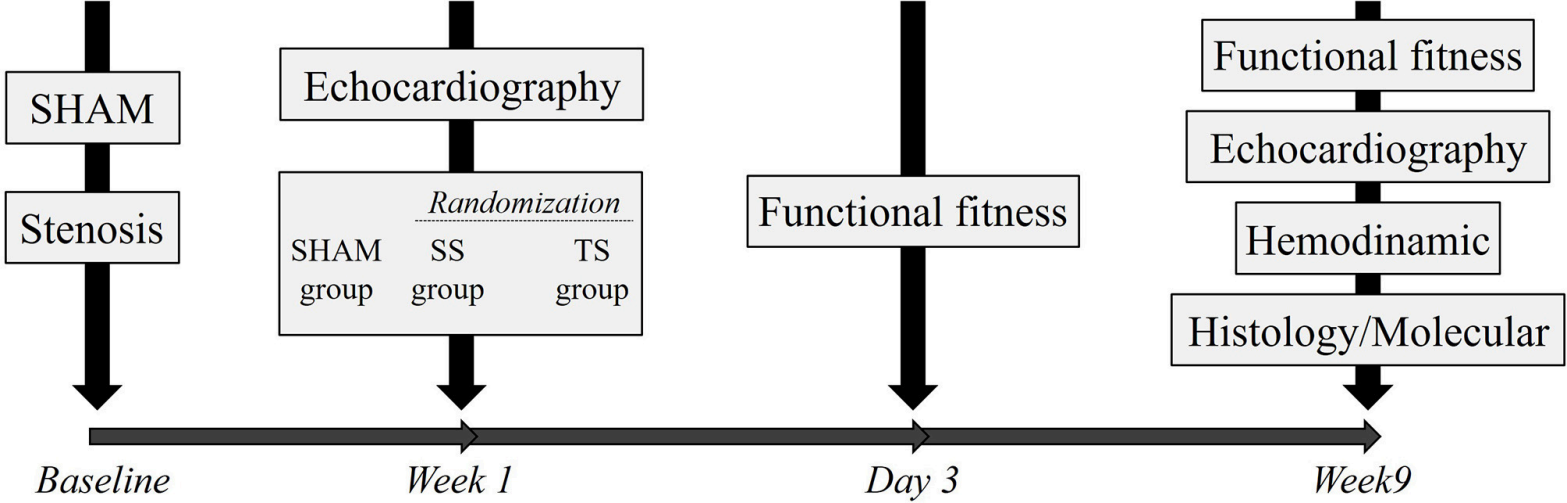
**Experimental design**.

The investigation complies with the Guide for the Care and Use of Laboratory Animals published by the US National Institutes of Health (NIH publication no. 85-23, revised 1996). The experimental protocol was approved by the Institutional Research Ethics Committee from the Federal University of São Paulo, São Paulo, Brazil (process: 1673020414). Sixty-three female Wistar rats, weighing 180–220 g and aged 8–9 weeks, were assigned to one of the following three groups: No-trained Sham (*n* = 21); no-trained stenosis (SS, *n* = 21); and trained stenosis (TS, *n* = 21). Figure 1 illustrates the experimental design, in which at first step the animals were submitted to stenosis or Sham procedure. A week later, echocardiographic examination was performed in all animals, and stenosis rats were then randomly assigned to SS or TS group. Following 3 days, the animals were submitted to functional fitness test on a motor-driven treadmill. Then, animals were followed for over 8 weeks.

### PAS surgery

Rats were anaesthetized with ketamine plus xylazine (50 mg/Kg, Dopalen®, Vertbrands, Paulínia, SP, BRA, i.p.) mixture, intubated, and mechanically ventilated (Rodent ventilator, model 683, Harvard Apparatus, Holliston, MA, USA). A left thoracotomy was performed, and the pulmonary artery (PA) was carefully dissected free from the aorta. A silk thread was positioned under the PA, and an 18-gauge needle was placed alongside the PA. A suture was tied tightly around the needle, and the needle was rapidly removed to produce a fixed constricted opening in the lumen equal to the needle diameter. The combination of a fixed banding around the PA and the animal growth resulted in a high RV afterload. The Sham animals underwent the same procedure except PAS (Faber et al., 2006).

### Functional fitness assessment (maximal oxygen uptake, VO_2_max)

The functional fitness was evaluated using a motorized treadmill coupled to a gas analyzer (Panlab, Harvard Bioscience Company, MA, USA). Prior to the physical test, rats were introduced to running as previously described by our group (Amadio et al., 2015). Each rat had to undergo a 2-min warm-up period at 25 cm/s, and the running speed was increased by 9 cm/s every 2 min to reach exhaustion. An oxygen uptake steady state on progressive increases in running speed and a respiratory exchange ratio of ≥1.05 were taken into account to define the VO_2_max.

### Echocardiography

After 48 h of stenosis or Sham surgery, rats were anaesthetized with ketamine and xylazine mixture as reported above, and transthoracic echocardiography was performed to determine pressure gradient using a 12 MHz transducer Sonos-5500 (Hewlett-Packard, Andover, MA, USA). The echocardiography was also carried out 9 weeks later to RV morphofunctional study. Measurements were performed according to the echocardiographic RV guidelines. The 2D modality was used to measure the RV. The right parasternal short-axis view at the level of the papillary muscles was used for determination of the diastolic and systolic RV area. Measurements of the RV outflow tract were obtained from the parasternal short-axis view at the level of the aortic valve during end-diastole (Egemnazarov et al., 2015). We included only animals with pressure gradients between 25–55 mmHg (Manchini et al., 2014). The diastolic (RVDA) and systolic (RVSA) transverse areas of the RV were measured at the basal, middle, and apical view. The final value was the arithmetic mean of the measures of the three views. Systolic function was analyzed by the fractional area change (FAC) as a function for the following equation: FAC = RVDA-RVSA/RVDA × 100. Pulsed Doppler at the tricuspid valve level provided the flow velocity curve to analyze the diastolic function (E/A wave ratio).

### Exercise training protocol

Rats were subjected to running training on a motor-driven treadmill (CL4002, Caloi, São Paulo, Brazil) after 7 days of surgical procedures for 8 weeks. Rats ran 6 times a week, and each session lasted up to 60 min. For each session, the treadmill speed was 18 m/min for 30 min and 22 m/min for the remaining 30 min. This research protocol has shown to be effective in alleviating the pathological cardiac remodeling (Serra et al., 2008, 2010).

### Myocardial growth markers and fibrosis

Twenty-four hours after the last training session or sedentary status the rats were anesthetized with urethane overdose (4.8 g kg kg^−1^ i.p.) and the hearts were quickly removed. The RV chamber was cut, separated from the rest of the heart and weighted to be used as a myocardial hypertrophy indicator. Afterwards, RV tissue was transversally sectioned at the mid-RV level and fixed in 10% formalin buffered solution. Tissue samples were sectioned in 7 μm thickness and stained with hematoxylin-eosin for cross-sectional cardiomyocyte area analysis at approximately 20 visual fields for each animal. Myocytes with visible nuclei and intact cellular membranes were chosen for evaluation. The analyses were performed at 40× magnification using an Olympus image acquisition system (Waltham, MA, USA) (Soci et al., 2011). The RV fibrosis was evaluated in tissue samples stained with *picro-sirius* red. The collagen content was assessed at 40× magnification using an Olympus image acquisition system (Egemnazarov et al., 2015). The percentage fibrosis was calculated by dividing the total area of collagen by the total analyzed tissue area multiplied by 100.

### mRNA assay

Total RNA was extracted from frozen RV with 1 ml of TRIzol reagent (Gibco BRL, MD, USA). RNA quantification was determined using a SpectraMax M5 spectrophotometer system (Molecular Devices, CA, USA). One microgram of RNA was used for cDNA synthesis and Real-Time PCR gene expression analysis. First, contaminating DNA was removed using DNase I (Invitrogen, CA, USA) at a concentration of 1 unit/μg RNA in the presence of 20 mM Tris-HCl, pH 8.4, containing 2 mM MgCl_2_ for 15 min at 37°C, followed by incubation at 95°C for 5 min. Then, the reverse transcription was performed with 200 μl reaction in the presence of 50 Mm Tris-HCl, pH 8.3, 3 mM MgCl_2_, 10 mM dithiothreitol, 0.5 mM dNTPs, and 50 ng of primers with 200 units of Moloney murine leukemia virus-reverse transcriptase (Invitrogen). The conditions were: 20°C for 10 min; 42°C for 45 min; 95°C for 5 min. One microliter of reverse transcription reaction was used for real-Time PCR at Applied Biosystems 7500 Fast PCR (ABI Prism, Applied Biosystems, CA, USA) using the SYBR Green supermix (ABI Prism). Experiments were performed in triplicates for each target genes: β-MHC (primers forward 5′-AAGTGGGCAGCATCACCTAC-3′ reverse 5′-GCCGGCTCTGTAACTTCCTT-3′ (GenBank: NM_021838); α-MHC (primers forward 5′-TCAGGCTTGGGTCTTGTTAGC GAAGAGAAACTTCCAGGGGCA-3′ reverse 5′-AGGCTC TTTCTGCTGGACA-3′ (GenBank: NM_012611.3). Target gene abundance was quantified as a relative value for internal control β-Actin: forward primer 5′-AGAGGGAAATCGTGCGTGAC-3′ and reverse primer 5′-AGGAAGGAAGGCTGGAAGAGA -3′ (GenBank: NM_031144.3).

### MicroRNA assay

The cDNA for miRNA analysis was synthesized from total RNA using specific primers according to the TaqMan microRNA assay. The 15 μl reactions obtained by the TaqMan MicroRNA Reverse Transcription Kit protocol (Applied Biosystems, CA, USA) were incubated in a Thermal Cycler (Applied Biosystems, CA, USA) for 30 min at 16°C, 30 min at 42°C, and 5 min at 85°C. They were then maintained steadily at 4°C. The real-time PCR quantification was performed by using the TaqMan MicroRNA Assay protocol (Applied Biosystems, CA, USA). The 20 μl PCR reaction solution contained 10 μl TaqMan Universal PCR master mix II (2x), 1.33 μl RT product, 7.67 μl nuclease-free water, and 1 μl of primers and probe mix from the TaqMan MicroRNA Assay protocol for microRNA-1 and 21. The reactions were performed at 95°C for 10 min, and then in 40 cycles of 95°C for 15 s and 60°C for 1 min. Samples were normalized by evaluating the U6 gene.

### Western blot

Frozen RV was homogenized using ice-cold lysis buffer and proteinase inhibitor cocktail (Manchini et al., 2014). Lysates corresponding to 30 μg of protein were subjected to 10% SDS-PAGE. Separated proteins were transferred to PVDF membrane (Amersham Biosciences, NJ, USA) and transfer effectiveness was examined with 0.5% Ponceau S. After blocking with 5% non-fat dry milk for 2 h at room temperature, PVDF membranes were probed with Abcam (Cambridge, MA, USA) primary antibodies for rabbit ant-Akt_1_ (1:5000), rabbit anti-p-Akt_1_ (1:2500), rabbit anti-Caspase3; rabbit anti-Bax (1:1000), rabbit anti-Bcl-2 (1:1000), rabbit anti-Bcl-xL (1:500), rabbit anti-β-MHC (1:5000), rabbit anti-α-MHC (1:5000), rabbit anti-L-type Ca^++^ (1:500), rabbit anti-ryanodine receptor (1:1000), rabbit anti-Serca 2 (1:1000), and rabbit anti-Na^+^/Ca^++^ exchanger (1:100) in overnight incubation. Membranes were then washed five times with PBS and incubated for 1 h with horseradish peroxidase-conjugated goat anti-rabbit (1:20,000; Zymed, CA, USA). Membranes were again washed five times with blocking buffer and then rinsed twice with PBS. Antibodies binding were detected by chemiluminescence reagents (Amersham Biosciences, NJ, USA), and images were captured using an Amersham Imager 600 system. Quantification of target proteins was normalized for the internal control glyceraldehyde 3-phosphate dehydrogenase.

### Statistical analysis

Data were analyzed using GraphPad Prism software version 5.0 (La Jolla, CA, USA) by analysis normality with Shapiro-Wilk test. One-way ANOVA complemented by Newman–Keuls *post-hoc* was used to detect differences between groups for cross-section analysis. Two-way repeated ANOVA complemented by Bonferroni *post-hoc* were applied to paired data. Kruskal-Wallis followed by Dunn's multiple comparison tests were applied to non-normality data. Statistical significance was set at *p*-value ≤0.05. Data are expressed as mean ± standard error of the mean.

## Results

### Overall characteristics and functional fitness of animals

**Figure 2:**
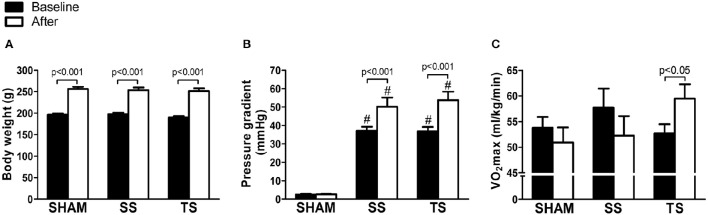
**Exercise training improves functional fitness in animals with PAS**. Data are representative of eight samples from each group in the baseline (filled columns) and at the end of the study (open columns). **(A)** Body weight. **(B)** Pressure gradient; **(C)** maximal oxygen uptake (VO_2_max). ^#^*p* < 0.001 vs. SHAM group for the respective time.

All rats survived the stenosis surgery or sham procedure. During the stenosis period, the rats did not show signs of cyanosis or congestion, as demonstrated by the similar liver (SHAM: 68.1 ± 3.5; SS: 66.1 ± 2.9; TS: 67.1 ± 3.6; %, *p* = 0.3) and lung (SHAM: 71.4 ± 4.7; SS: 71.7 ± 5.2; TS: 71.6 ± 8.6; %, *p* = 0.9) weights. We performed the experiments in female rats because it allowed a strict control body weight. It is true that the stenosis degree could result in very dissimilar RV afterload during the study, thereby a bias would be set. As illustrated in Figure 2A, all experimental groups showed a similar average body weight at baseline and at the end of the study. The increase in body weight was associated with significant elevation in pulmonary transvalvular gradient only in stenosis groups (Figure 2B). This finding shows that the construction of the outflow tract of the RV was kept during the observation period, and echocardiography showed that the stenosis degree was similar between SS and TS groups. Moreover, mean valvar gradient values are within a range of mild to moderate stenosis definition (Hirth et al., 2006), which usually receives only clinical monitoring of RV remodeling. This issue emerges as a main way of analyzing the impact of exercise because critical stenosis requires a more invasive intervention (Godart et al., 2014). The Figure 2 also shows the functional fitness data. The VO_2_max was not significantly different between groups at the baseline and at the end of the study, but there was a slight decrease in the SHAM and SS groups after 8 weeks (Figure 2C). A single functionality difference was evident only in the TT rats, in which VO_2_max was significantly increased with training.

### Exercise prevents concentric RV remodeling and fibrosis

**Figure 3:**
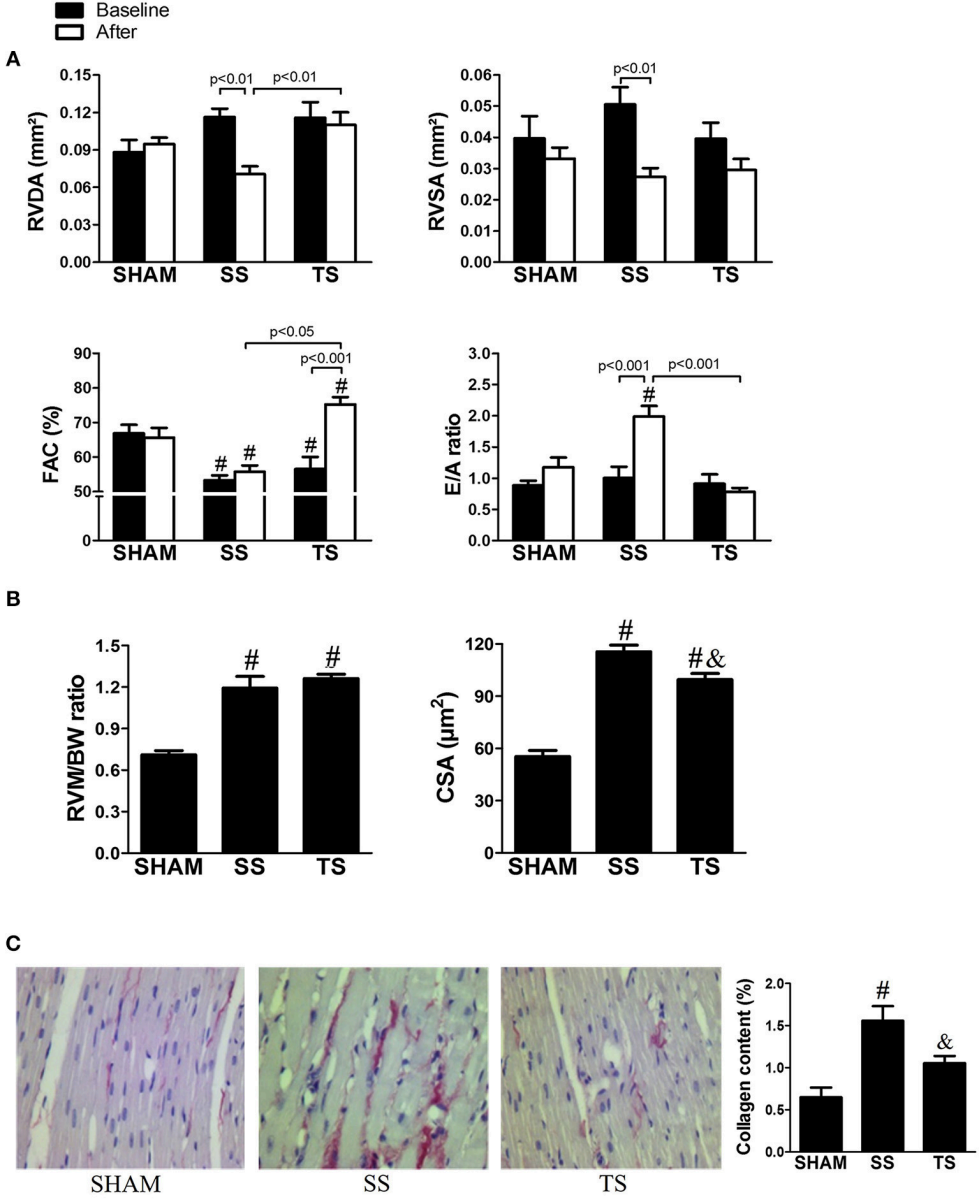
**Effects of exercise training on right ventricular hypertrophy and fibrosis induced by PAS. (A)** Echocardiographic analysis of eight samples from each group in the baseline (filled columns) and at the end of the study (open columns): RVDA, right ventricular diastolic area; RVSA, right ventricular systolic area; FAC, fractional area change; E/A ratio, ratio between the E and A waves. **(B)** RVMBW, right ventricular mass/body weight (SHAM, *n* = 5; SS, *n* = 6; TS, *n* = 6); CCA, cross-sectional cardiomyocyte area (SHAM, *n* = 3; SS, *n* = 4; TS, *n* = 5); **(C)** Representative photomicrographs of four samples from each group showing myocardial collagen on magnification ×40 (Scale bar, 250 μm). ^#^*p* < 0.001 vs. SHAM group for the respective time. ^&^*p* < 0.05 vs. SS group.

On transthoracic echocardiography (Figure 3A), we have confirmed previous findings from the literature that the RV-pressure overload results in a concentric cardiac remodeling (Faber et al., 2006). From the baseline to end of the study, non-trained stenosis rats (SS group) had a significant reduction in diastolic and systolic RV transverse areas, characterizing a concentric RV remodeling. On the other hand, exercise prevented these concentric changes in the cavity. The echocardiographic analysis also revealed reduced RV systolic performance in the first analyzes for animals submitted to stenosis. An evaluation 8 weeks after operation illustrated that systolic dysfunction was stable in SS group, but a significant improvement was noted in the animals submitted to exercise (TS group). The stenosis rats exhibited an increased ratio of RV weight to body weight and cross-section cardiomyocyte area compared with SHAM rats, in which trained animals showed a lower cross-section cardiomyocyte area compared to SS group (Figure 3B). Our structural analysis corroborates a well-established finding of myocardial fibrosis associated with RV pressure overload (Baicu et al., 2012; Egemnazarov et al., 2015). Thereby, stenosis resulted in a significant increase in collagen content (Figure 3C). Notably, exercise prevents the collagen deposition, in which TS group had values that did not differ significantly from the SHAM group.

### Exercise triggers survival pathway and inhibits myocardial apoptosis

**Figure 4:**
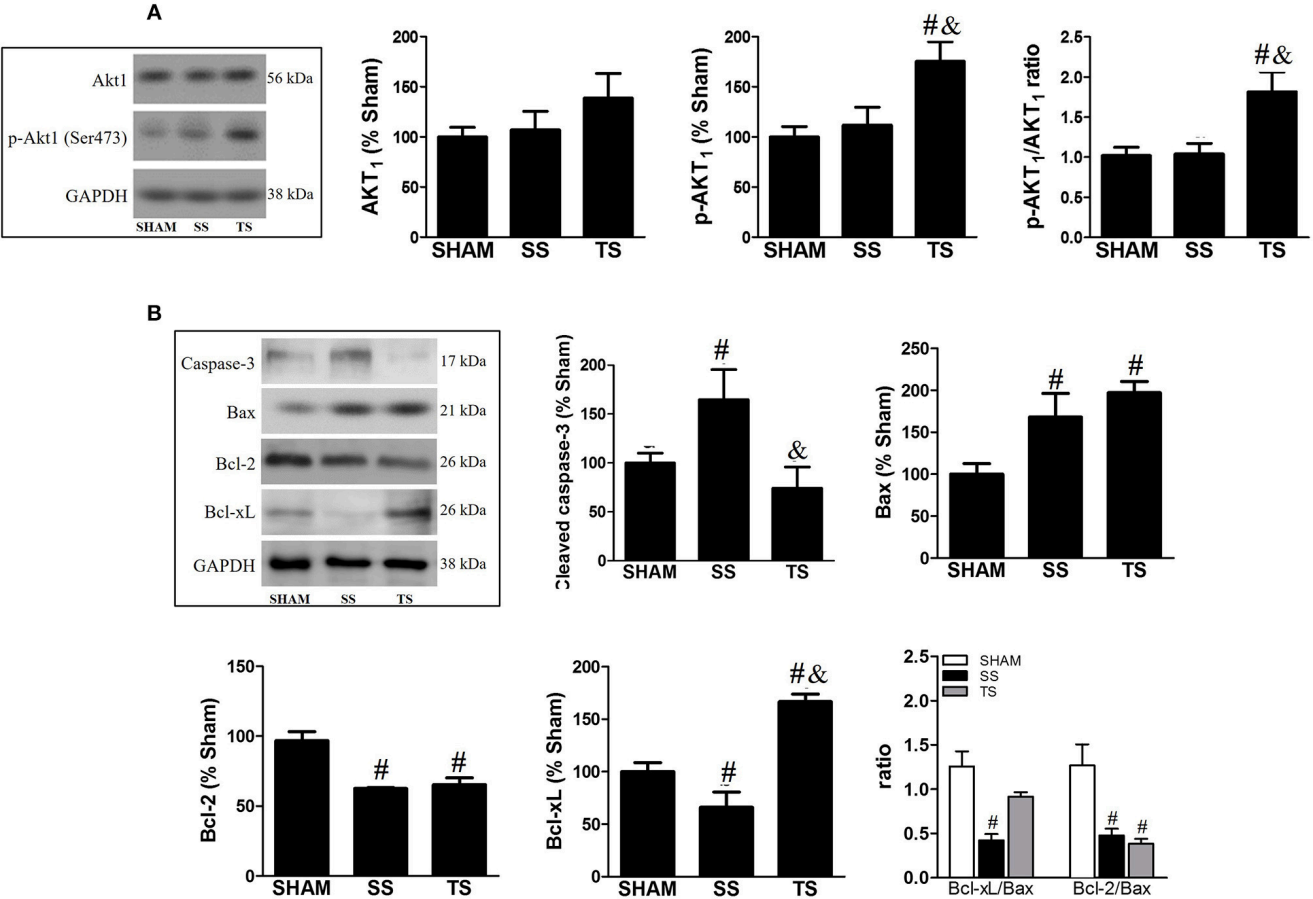
**Exercise training modulates key components of the survival and mitochondrial apoptosis pathway in the RV myocardial on PAS**. It is remarkable that activated form of Akt_1_ (**A:** SHAM, *n* = 8; SS, *n* = 8; TS, *n* = 7) and anti-apoptotic Bcl-xL (**B:** SHAM, *n* = 8; SS, *n* = 6; TS, *n* = 7) were significantly up-regulated in the exercise animals. Moreover, a beneficial effect of exercise was observed for cleaved caspase-3 protein **(B)**, in which indicates a myocardial apoptosis normalization. ^#^*p* < 0.05 vs. SHAM group. ^&^*p* < 0.01 vs. SS group.

We analyzed the Akt_1_ and p-Akt_1_ expression, as altering its activity provides a potent pro-survival signal (Matsui et al., 2003). As illustrated in Figure 4A, there was a significant upregulation of the activated Akt_1_ form only in trained animals. We further investigated whether the increase in Akt_1_ activity could be associated with a beneficial role of exercise in cell death. Thus, RV apoptosis was assessed by cleaved Caspase-3 levels, which was significantly increased in PAS animals (Figure 4B). These findings were accomplished by significant changes in upstream components of myocardial mitochondrial-dependent apoptotic signaling pathways. Compared to SHAM group, the protein pro-apoptotic Bax level significantly increased while the anti-apoptotic factors Bcl-2 and Bcl-xL decreased in the SS and TS groups. Intriguingly, exercise restored the cleaved Caspase-3 to a similar level of SHAM animals. These findings were accomplished by a significant increase of anti-apoptotic Bcl-xL. Hence, exercise training reversed to normal the Bcl-xL/Bax ratio in myocardial.

### Myocardial MicroRNA-1 and -21 levels are regulated by exercise

**Figure 5:**
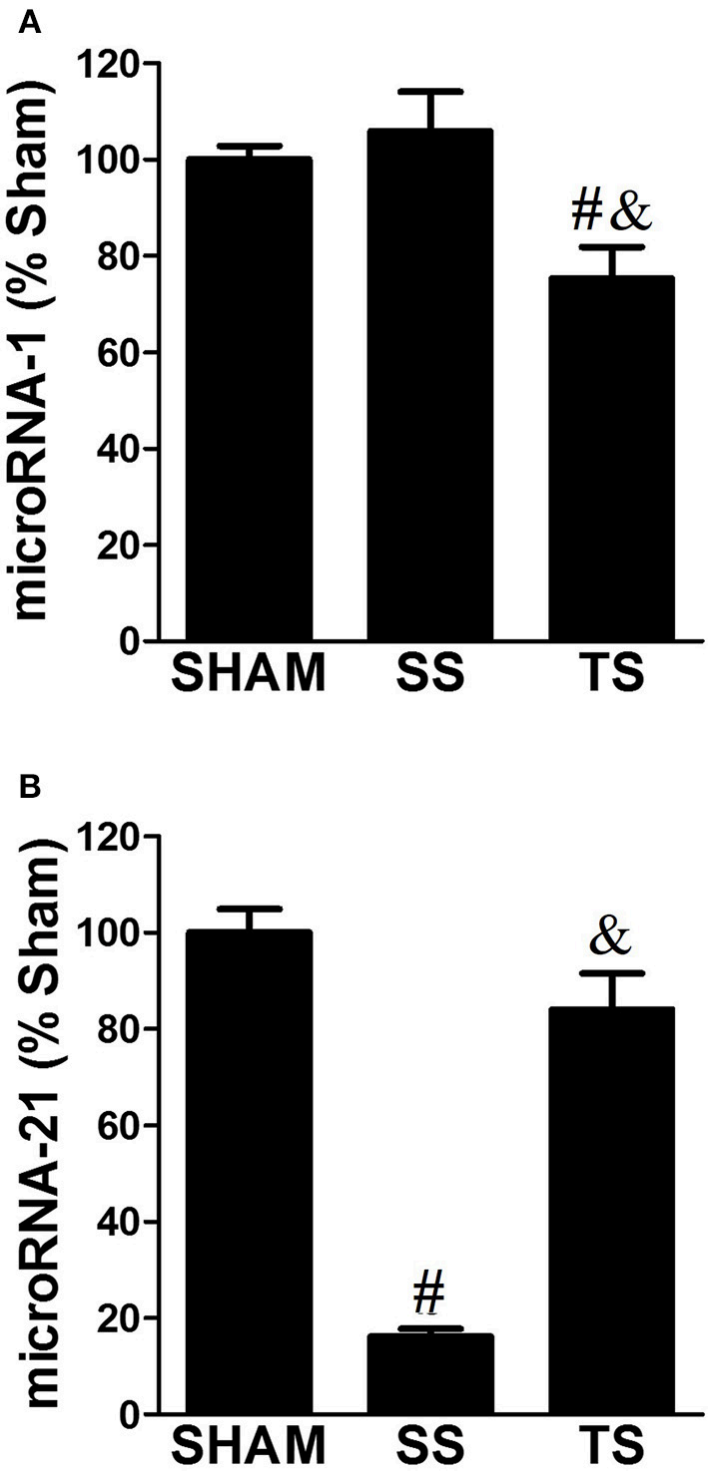
**Exercise training decreases microRNA-1 (A)** and normalizes microRNA-21 **(B)** myocardial expression in PAS animals (SHAM, *n* = 4; SS, *n* = 6; TS, *n* = 6). All values were normalized for levels of U6 gene. ^#^*p* < 0.001 vs. SHAM group. ^&^*p* < 0.001 vs. SS group.

MicroRNAs are emerging as key regulators of gene expression, and their role in cardiac hypertrophy is becoming increasingly apparent (Harada et al., 2014). When looking at the expression of microRNA-1 we found no effect of pulmonary stenosis *per se*, but there was a significant reduction with exercise (Figure 5A). We observed a strong effect of stenosis on the microRNA-21 expression, in which was down-regulated in the SS group (Figure 5B). On the other hand, a restored microRNA-21 level was reported with exercise.

### Exercise modulates myocardial gene/protein isoform switches

**Figure 6:**
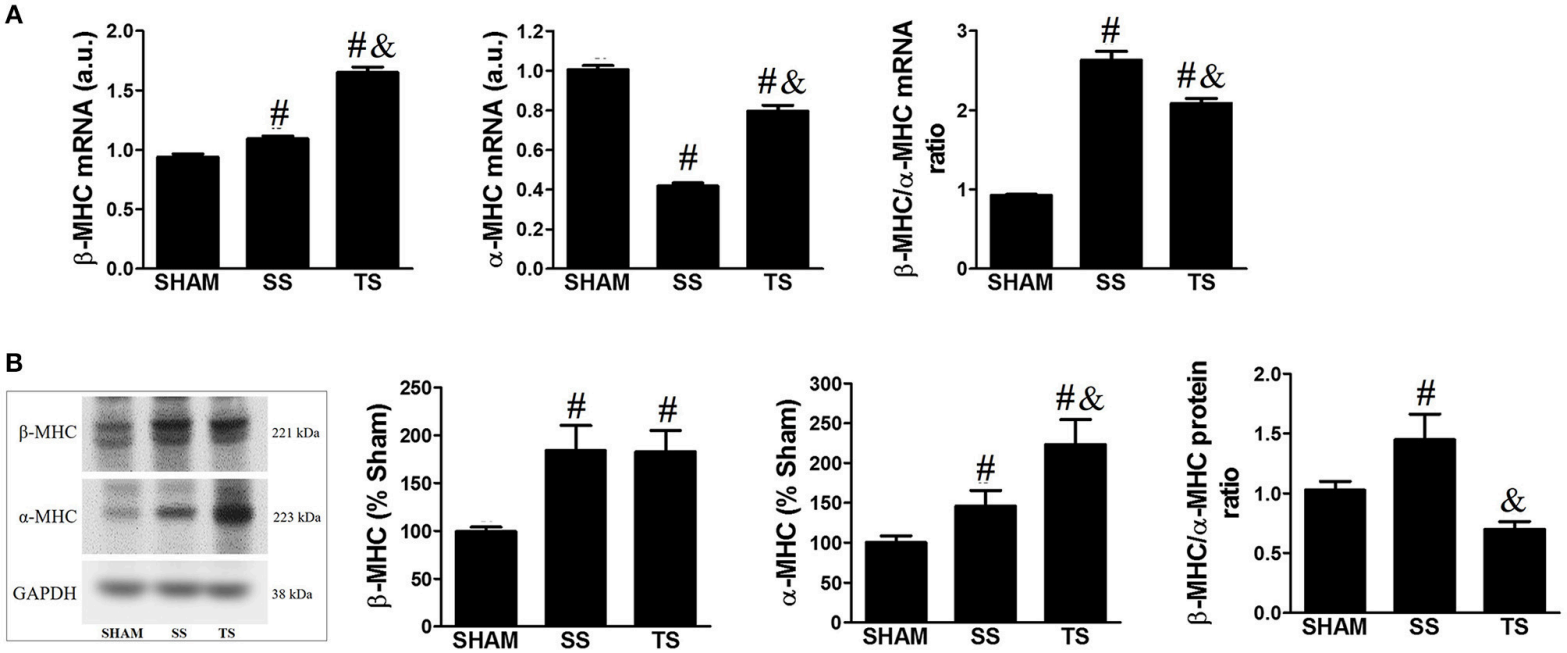
**Exercise training modulates β-MHC and α-MHC isoform expression in myocardial. (A)** Gene expression was evaluated by real-time PCR (SHAM, *n* = 4; SS, *n* = 6; TS, *n* = 6). **(B)** Protein expression was evaluated by Western blot (SHAM, *n* = 7; SS, *n* = 6; TS, *n* = 7). Values were normalized for levels of glyceraldehyde-3-phosphate dehydrogenase (GAPDH). ^#^*p* < 0.05 vs. SHAM group. ^&^*p* < 0.01 vs. SS group.

We assessed how the pathophysiologic modifications in the RV were reflected by gene/protein expression changes of two distinct types of myosin heavy chain (MHC), referred as β-MHC and α-MHC. These analyses were carried out because the relative distribution of β- and α-MHC is altered in cardiac hypertrophy and showed to be directly correlated with the myocardial dysfunction (Nadal-Ginard and Mahdavi, 1989). Consistent with myocardial remodeling, there was increased β-MHC and decreased α-MHC gene expression in stenosis animals (Figure 6A). However, the combination of the stenosis with the exercise resulted in additional expression of the β-MHC and attenuation in downregulation of α-MHC. Thereby, the β/α-MHC ratio was increased in SS group whereas this was attenuated by exercise. The Figure 6B illustrates that β-MHC and α-MHC protein expression was upregulated for both SS and TS groups. Moreover, stenosis-induced overexpression of α-MHC protein was significantly enhanced by exercise. Then, the increased β/α-MHC ratio induced by stenosis was completely abrogated in trained animals.

### Exercise modulates expression of Ca^++^-regulating proteins

**Figure 7:**
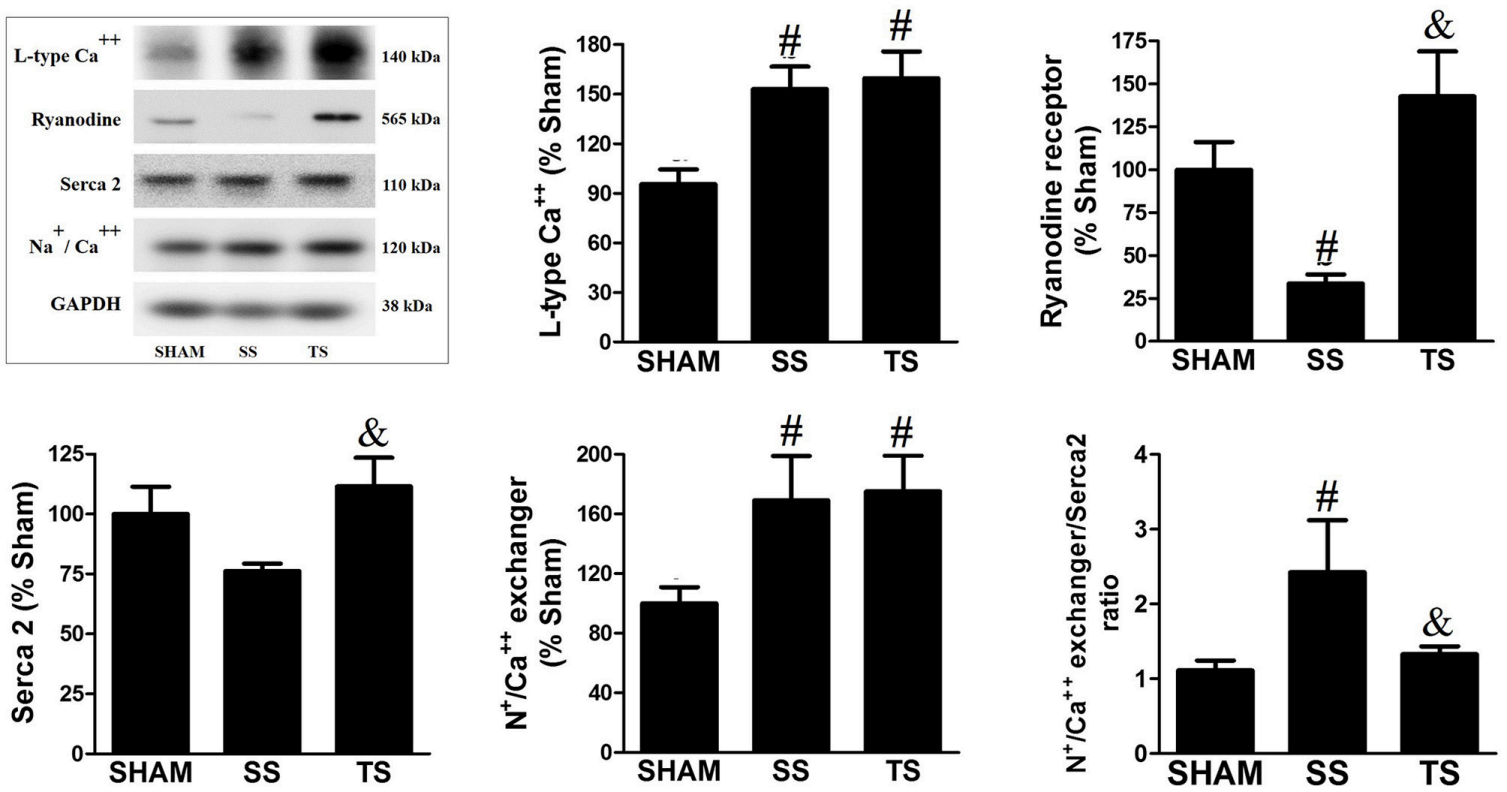
**Exercise training modulates myocardial calcium handling protein levels in PAS animals (SHAM, ***n*** = 8; SS, ***n*** = 8; TS, ***n*** = 7)**. Values were normalized for levels of glyceraldehyde-3-phosphate dehydrogenase (GAPDH). ^#^*p* < 0.05 vs. SHAM group. ^&^*p* < 0.05 vs. SS group.

Quantitative changes in expression of the proteins that modulate calcium handling with correlations to functional alterations were reported in several experimental models of cardiac disease (Wankerl and Schwartz, 1955). Here, pulmonary stenosis induced a significant increase in L-type Ca^++^ channel and as Na^+^/Ca^++^ exchanger expression (Figure 7). Furthermore, SS animals exhibited lower ryanodine receptor content and a no significant reduction of Serca 2. Exercise protocol restored both ryanodine receptor and Serca 2 expression to basal levels. In addition, our results demonstrate an imbalance of Na^+^/Ca^++^ exchanger and Serca 2 expression in SS animals, while TS group has not reported this finding.

## Discussion

In this study, we showed for the first time a useful role of exercise in a major congenital malformation affecting the RV outflow, i.e., PAS. Previous studies have shown only beneficial effects of exercise on RV remodeling in pulmonary hypertension model (Souza-Rabbo et al., 2008; Colombo et al., 2013). We have well-ordered the body weight of the animals at baseline to ensure a comparable stenosis level between experimental groups, thereby imposing similar RV overload (Figure 2B).

We have observed RV pressure gradients ranging ~40 to 50 mmHg/s, and this led to RV hypertrophy and remodeling, as reflected by increased RV mass, increased cardiomyocyte cross-sectional area, a concentric pattern of the RV, and fibrosis. As previously described by other authors (Egemnazarov et al., 2015), myocardial remodeling was associated with reactivation of the “fetal gene program,” as shown by the shift from α-MHC to β-MHC expression. Thus, as seen in Figure 4, the β-MHC to α-MHC ratio was shifted upwards in transcriptional and translational level. To date, although systolic and diastolic dysfunction has been reported in our PAS model, we did not observe signs of cardiac failure, as shown by the lack of cyanosis and liver or lung congestion. These findings are in line with interpretations from other groups (Johnson et al., 2011; Egemnazarov et al., 2015) and emerged as a possibility to explain because no significant reduction in functional capacity was noticed with PAS (Figure 2C).

To our knowledge, no published studies have directly assessed the role of exercise on RV hypertrophy induced by PAS. There is only information showing that an exercise program may be beneficial in other RV pressure overloads. In a pulmonary hypertension model induced by monocrotaline, Colombo et al. (2013) showed that hypertensive-exercised animals exhibited reduction in collagen, increase in vessels, and attenuation of diastolic dysfunction when compared with the hypertensive-untrained animals. A more recent study showed that exercise was capable of attenuating RV hypertrophy, improving cardiac function, and increasing exercise tolerance and survival in rats with pulmonary hypertension (Moreira-Gonçalves et al., 2015). Here, we have reported that exercise exerts several beneficial effects on RV remodeling induced by PAS. Our histomorphometric data show that exercised-stenosis animals had lower cellular hypertrophy and fibrosis. Moreover, the echocardiographic analysis revealed a cardioprotective action of exercise on RV architecture and function, i.e., there was prevention of concentric cavity pattern, improved systolic performance and conserved diastolic function. Thus, it may be suggested that TS animals exhibited a prototype of physiological myocardial stimulus associated to preserved cavity geometry and improved cardiac function (Ellison et al., 2012). These benefits occurred regardless of a reduction in gradient pressure, which supports the hypothesis that the exercise can attenuate myocardial remodeling independent of the cardiac overload lowering the effects of exercise (Libonati, 2013). Overall, benefits from the exercise may explain the improved functional capacity in trained animals, as noted for the increase in VO_2_max—a gold standard to assess exercise tolerance (Carlson, 1955). Therefore, considering that, a poor exercise tolerance is usually seen in patients with RV hypertrophy or who had surgical repair (Roos-Hesselink et al., 2006; Meadows et al., 2007), exercise can be an attractive approach.

We examined RV histological and molecular features to provide insights into the exercise-induced cardioprotection. Exercise animals showed minor cardiomyocyte hypertrophy with fibrosis inhibition, which can be linked to restoring the diastolic function (E/A ratio) as a result of myocardial stiffness normalization. Moreover, maintenance of RV cavity dimension and minor cross-sectional area of cardiomyocytes can be related to increased activity of Akt_1_. In fact, some studies demonstrate that the Akt activation has a favorable impact on cardiac pressure overload by inhibiting or preventing pathological processes (Faber et al., 2006; McMullen et al., 2007; Harston et al., 2011). Considering that the Akt is one of the major upstream signals of the Bcl-2 family, a higher activity of Akt can extend to the inhibition of broader cascade of apoptosis in trained animals. In fact, exercise training inhibited the increase of key downstream effector—cleaved Caspase-3, and more than normalized levels of anti-apoptotic factor Bcl-xL. This information is in line with current literature, in which exercise is shown to be a useful approach to prevent heart apoptosis caused by an increased afterload (Huang et al., 2012).

We also tried to explore the expression of two well-documented microRNAs to respond to hypertrophic stimuli. It can be observed that mirRNA-1 was unchanged in the untrained animals. These findings are surprising because it was presumed that the microRNA-1 levels could be reduced with PAS, as previously reported in rodents subjected to aortic binding (Wang et al., 2009). However, microRNA-1 was reduced only in trained animals, showing that the physiological exercise stimulus persists even with pathological PAS insult. Previous studies have identified similar data (Wang et al., 2009; Ellison et al., 2012), but the physiological impact of altered mirRNA-1 level still needs clarification in healthy and diseased heart. Further, microRNA-21 expression was found to be significantly lower in SS animals, and these findings against previous studies showing upregulation of microRNA-21 in response to aortic binding. In this regard, microRNA-21 ablation has been advocated as a beneficial therapeutic to cardiac remodeling. Thum et al. (2008) reported that microRNA-21 knockdown prevented cardiac hypertrophy and fibrosis in response to pressure overload. The simple interpretation of these findings could indicate that the increase in microRNA-21 with exercise would not provide for myocardial remodeling. Nevertheless, some issues show to be clarified: (i) Is microRNA-21 upregulated in both ventricles in response to chronic elevated afterload? (ii) How can microRNA-21 ablation caused by exercise affect the RV remodeling? (iii) The maintenance of myocardial microRNA-21 level in SHAM and TS groups leads us to believe that the microRNA-21 works to promote cell survival through inhibition of PTEN (phosphatase and tensin homolog deleted on chromosome ten). The PTEN degrades phosphatidylinositol-3,4,5-trisphosphate, which is produced by phosphoinositide 3-kinase and is essential for activation of the pro-survival Akt pathway (Kukreja et al., 2011). This issue was well reported in studies showing that the PI3K inhibitor abrogated the protective miR-21 effect on myocardial apoptosis (Tu et al., 2013). Thus, it is possible that steady-state microRNA-21 level can mediate inhibition of apoptosis induced by PAS in our trained animals. This assumption has not been explored.

It has also been indicated that exercise more than restored systolic RV function in PAS animals. It is reasonable to admit that apoptosis inhibition resulted in conserved cardiomyocyte amount in the TS animals, contributing to preserving RV function. We have reported changes in the α-MHC and β-MHC composition in transcriptional e translational level. Considering that, an increased α-MHC expression, which has high Ca^++^ and actin-activated ATPase activity (Nadal-Ginard and Mahdavi, 1989), it is possible that the increase in α-MHC mediates improved systolic function in the TS group. Moreover, the slight increase of α-MHC can counteract the increased β-MHC expression, which has lower ATPase activity. Besides, exercise showed to modulate the altered Ca^++^-regulating proteins expression induced by PAS. Although the SS and TS groups have shown similar increase in L-type Ca^++^ channel density (Figure 7), which can trigger a high amount of Ca^++^ from the Serca 2 (Balke and Shorofsky, 1998), the reduced ryanodine density could decrease the gain of Ca^++^-induced Ca^++^ in the SS group. This finding was well documented in studies carried out mice with reduced ryanodine receptor, in which Ca^++^ release from sarcoplasmic reticulum was impaired in isolated cardiomyocytes (Zou et al., 2011). In addition, Serca 2 tended to be depressed and Na^+^/Ca^++^ exchanger/Serca 2 ratio was increased in RV from SS animals. We did not directly assess the Ca^++^ uptake function of the Serca 2 or the impact of increased Na^+^/Ca^++^ exchanger, but it can be assumed that these findings will slow the decline in [Ca^++^]i during relaxation and/or cause a net loss of Ca^++^ due to alternative removal via NCX (Bers et al., 2002). On the other hand, this condition could not exist in the trained animals, in which ryanodine receptor and Serca 2 densities as well as Na^+^/Ca^++^ exchanger/Serca 2 ratio were restored.

In summary, the major outcome of this study was to show that exercise training attenuates myocardial remodeling and improves RV function as well as functional fitness in rats with PAS. These changes were found to be associated with preserved collagen content and apoptosis in myocardial. The cardioprotective role of exercise may be caused by positive modulation of sarcomeric protein and calcium handling protein levels. For clinical purposes, exercise training can be considered as a useful approach for several patients with increased RV afterload, whether from PAS, pulmonary arterial hypertension, or other cardiac disorders, without a well-defined RV failure until delayed clinical course. Therefore, exercise may be a key tool to the development of overt RV failure. Moreover, given that an increased RV afterload can result in biventricular injury and dysfunction (Okumura et al., 2015), it is possible that exercise benefits also exist in the left ventricle. Unfortunately, we have not carried out analysis of left ventricle and this will be evaluated in the future.

## Author contributions

BLM, SSV, ELA, LFNS, LP, and RL contributed to the design of the study, acquisition of data, and result analyses. HAO performed statistical analyses. JASJ and PTCC revised the manuscript, and approved the final version. PJFT and AJS raised grant funding, contributed to the design of the study, revised the manuscript, and approved the final version.

## Funding

This work was supported by the FAPESP on grant numbers 09-54225/8 and 2015/11028-9.

### Conflict of interest statement

The authors declare that the research was conducted in the absence of any commercial or financial relationships that could be construed as a potential conflict of interest.

## Abbreviations

RV: Right ventricle
PAS: Pulmonary arterial stenosis
SS: No-trained stenosis
TS: Trained stenosis
PA: Pulmonary artery
RVDA: Right ventricle diastolic transverse area
RVSA: Right ventricle systolic transverse area
FAC: Fraction area change
MHC: Myosin heavy chain.
